# Evaluation of a novel liquid-solid biphasic differential medium for screening of group B *streptococcus* in perinatal women

**DOI:** 10.1128/spectrum.00140-25

**Published:** 2025-05-13

**Authors:** Shuo Chen, Fangqu Li, Xiangyang Li, Hao Chen

**Affiliations:** 1 Department of Nosocomial Infection Prevention and Control, The First Affiliated Hospital of Wenzhou Medical University 89657, Wenzhou, Zhejiang Province, China; 2 Wenzhou Medical University 26453, Wenzhou, Zhejiang Province, China; 3 Department of Clinical Laboratory, The Second Affiliated Hospital and Yuying Children’s Hospital of Wenzhou Medical University 26453, Wenzhou, Zhejiang Province, China; City of Hope, Duarte, California, USA

**Keywords:** group B *streptococcus*, novel liquid-solid biphasic differential medium, blood agar plate

## Abstract

This study explored the capabilities of the commonly used blood agar plate in Chinese clinical microbiology laboratories and a novel liquid-solid biphasic differential medium (L-S StrepB) for detecting group B *streptococcus* (GBS) in peripartum women in China. A total of 11.2% (65/582) of pregnant women at 35–37 weeks of gestation were found to carry GBS. The sensitivity and specificity of L-S StrepB were 96.9% and 100%, respectively. Additionally, 87.3% (55/63) of GBS cases were detected within 15–24 h using L-S StrepB, demonstrating rapid processing time. This method is user-friendly, cost-effective, and requires no specialized equipment, making it suitable for use in primary healthcare settings.

**IMPORTANCE:**

The clinical application methods for group B *streptococcus* (GBS) detection among peripartum women in medical institutions in China are influenced by various factors, such as cost and personnel availability, leading to a lack of standardization. In this study, we evaluated a novel liquid-solid biphasic differential medium (L-S StrepB) that integrates liquid enrichment with solid agar for the detection of GBS. This is the first reported study in China to utilize biphasic medium for GBS detection. L-S StrepB demonstrates good sensitivity and specificity, with simple operations and low costs, while adhering to guidelines recommending initial enrichment followed by culturing.

## INTRODUCTION

*Streptococcus agalactiae*, also known as group B *streptococcus* (GBS), is a gram-positive β-hemolytic bacterium that acts as an opportunistic pathogen. GBS commonly asymptomatically colonizes the human gastrointestinal and genitourinary tracts and is recognized as a significant pathogen associated with perinatal infections in pregnant women and neonates (1, 2). GBS infection during the perinatal period can lead to a range of complications, including bacteremia, asymptomatic bacteriuria, preterm labor, premature rupture of membranes, amnionitis, and wound infections (3). In neonates, GBS infection can result in severe conditions such as sepsis, pneumonia, meningitis, and occasionally cellulitis (3, 4). The primary risk factor for GBS infection in neonates is transmission from the maternal colonization of the urogenital tract during the birthing process. The guidelines issued by the American College of Obstetricians and Gynecologists recommend screening for GBS in pregnant women at 36–37 weeks of gestation. Specimens should be collected using swabs from the vaginal-rectal area for GBS detection (5). Notably, the 2021 Chinese Expert Consensus endorses the recommendation for GBS screening at 35–37 weeks of gestation (6). The Centers for Disease Control and Prevention (CDC) recommends that if the test result is positive, the mother should typically receive beta-lactam antibiotics during labor to reduce the risk of transmission and subsequent disease (7).

Currently, the American Society for Microbiology considers culture to be the gold standard for the detection of GBS (8). To achieve increased detection, the guide recommends that all vaginal-rectal swabs should first be inoculated into a selective enrichment broth medium, followed by further culture or nucleic acid amplification testing (NAAT). Professionally trained personnel are needed to identify suspicious colonies when using the blood agar plate. The CDC also suggests the use of chromogenic medium to enhance recovery and simplify the identification of GBS during screening. However, the conventional broth enrichment method significantly increases the turnaround time, as the final result is not available until 48–72 h after the receipt of a vaginal-rectal sample (9).

Significant differences in maternal colonization rates have been reported based on region, ethnicity, and socioeconomic characteristics (10). Huang et al. (11) reported global rates of GBS colonization in pregnant women ranging from 2.0% to 32.0%. In the Americas, the GBS colonization rate among pregnant women was noted to be 18.3%, while it was 15.4% in Southern Europe and 11.0% in Asia (12). The overall colonization rate in China is notably lower than the global average of 17.4% (12–15). However, many of these are due to the low colonization rates resulting from unscientific screening methods (15, 16). The clinical application methods of GBS detection in Chinese medical institutions are affected by many factors, such as cost and personnel, and cannot be unified. However, as a populous country with an annual birth rate exceeding 10 million, China faces a substantial burden from infant invasive diseases attributable to maternal GBS colonization. Research has revealed an increasing burden and a remarkably high rate of multidrug resistance in GBS over the past decade (17). An analysis revealed invasive GBS disease in 0.55 per 1,000 live births in China, surpassing the global GBS incidence rate and being higher than previous estimates for Asia (18). These findings underscore the critical need for accurate screening for GBS and timely preventive treatment for pregnant women in China.

In this study, we evaluated a novel liquid-solid biphasic differential medium (L-S StrepB), a Granada-type medium with color-based visualization. This medium integrates liquid enrichment with solid agar cultivation for the detection of GBS, adhering to the recommended guidelines of prior enrichment followed by culturing. This method was compared with the blood agar plate commonly used in primary healthcare settings in China.

## MATERIALS AND METHODS

### Sample collection

Samples were collected from 582 pregnant women at 35–37 weeks of gestation who attended prenatal examination between July 2022 and October 2022 at the Second Affiliated Hospital and Yuying Children’s Hospital of Wenzhou Medical University. Dual vaginal/rectal swabs were obtained for each participant. Repeat participants were excluded from this study.

### Strain culture

All swabs were transported to the laboratory within 4 h post-collection. One swab was immediately inoculated onto a blood agar plate (Renfu Blocell, Zhengzhou, China), which was routinely used in our laboratory. Concurrently, the other swab was inoculated onto the liquid phase of L-S StrepB (Bacet, Wenzhou, China), which includes selective agents designed to inhibit or suppress the growth of enteric organisms and some gram-positive bacteria. After thorough rotation to ensure inoculation, the liquid was decanted onto the solid phase slope two to three times. The L-S StrepB was then placed on an orbital shaker for continuous shaking incubation. The blood agar plate and the L-S StrepB were maintained in an atmosphere of 5% CO_2_ at 35°C ± 2°C. To standardize and determine the analytical parameters of the methodology, specific reference strains were obtained from the American Type Culture Collection (ATCC) and the China General Microbiological Culture Collection Center (CMCC): *Streptococcus agalactiae* ATCC 12386 and *Streptococcus pyogenes* CMCC 32210.

### Result interpretation of L-S StrepB

The results were read at 15–24 h post-incubation. According to the manufacturer’s instructions, the presence of orange to orange-red colonies on the solid-phase slope and/or an orange-red coloration in the liquid phase was defined as GBS positive. If no color reaction was observed at the 15–24 h mark with L-S StrepB, the liquid was decanted onto the solid-phase slope two to three additional times and further incubated on the shaker until 48 h. The absence of bacterial growth or the presence of white or colorless colonies was interpreted as GBS negative.

### Strain identification

Suspected GBS colonies growing on blood agar plates were characterized as gray to white, translucent colonies with a narrow zone of β-hemolysis or non-hemolytic characteristics. These colonies were subsequently identified by matrix-assisted laser desorption ionization-time-of-flight mass spectrometry (MALDI-TOF MS) (Bruker Daltonics, Germany).

To further evaluate the accuracy of L-S StrepB in identifying GBS, orange-red colonies on the solid phase were identified using MALDI-TOF MS. If only the liquid phase of L-S StrepB exhibited a color change, the liquid was subcultured on the blood agar plate, followed by MALDI-TOF MS. Upon confirmation, all orange-red colonies on L-S StrepB were indeed identified as GBS.

**Fig 1:**
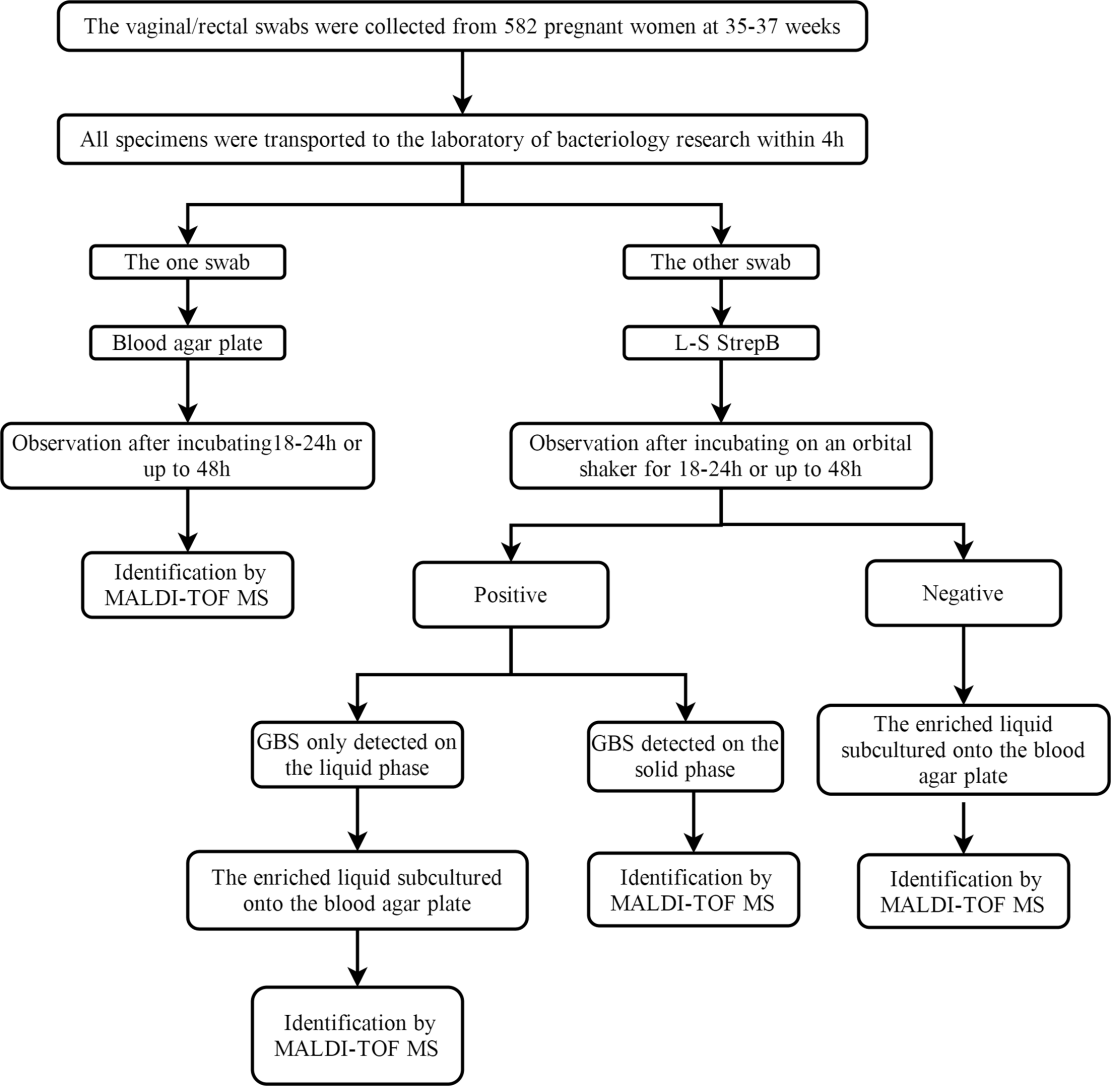
Overall study specimen selection and workflow.

Additionally, when the samples tested negative on L-S StrepB, the enriched liquid was subcultured onto a blood agar plate to evaluate potential false negatives, resulting in the detection of two GBS strains. This extra step was specifically designed to assess the accuracy of L-S StrepB and was not required in routine laboratory practice. Figure 1 outlines the workflow of the overall study specimens. Clearly, this additional step for identification also aligns with the guidelines that recommend initial enrichment followed by subsequent transfer for culturing (7).

### Statistical analysis

The sensitivity, specificity, positive predictive value (PPV), and negative predictive value (NPV) were calculated separately using the strains identified by both methods as references. All statistical analyses were performed using Statistical Package for the Social Sciences version 25.0. The Chi-square test was used to analyze categorical variables, and *P* < 0.05 was considered statistically significant.

## RESULTS

### Colony morphology of GBS on L-S StrepB

**Fig 2:**
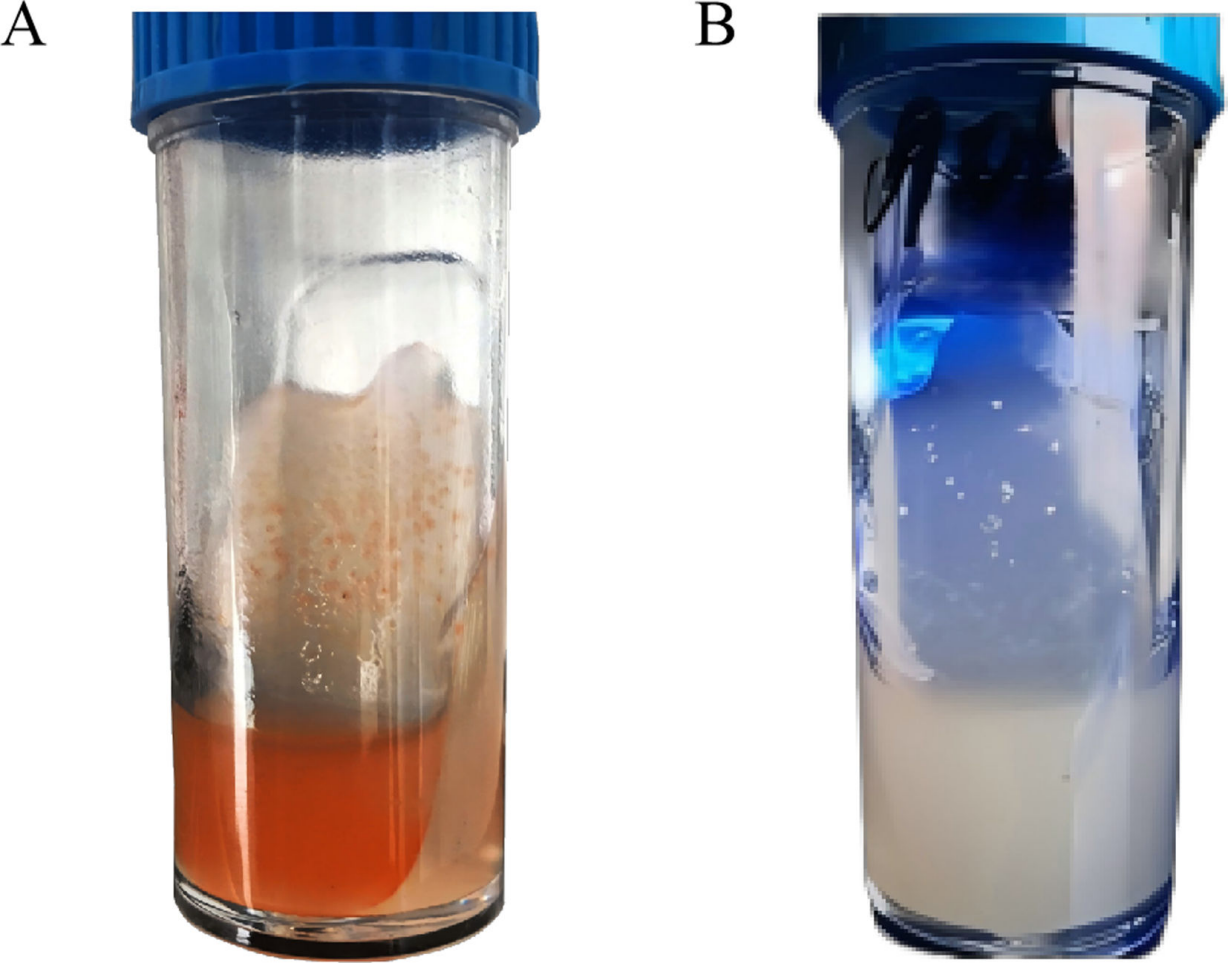
The colony morphology of GBS on L-S StrepB. (A) *Streptococcus agalactiae* ATCC 12386. (B) *Streptococcus pyogenes* CMCC 32210.

The specific reference strain, *Streptococcus agalactiae* ATCC 12386, formed orange to orange-red colonies greater than 0.5 mm on the solid phase slope of the L-S StrepB, with the liquid phase also exhibiting a corresponding orange-red color. In contrast, *Streptococcus pyogenes* CMCC 32210 produced colonies larger than 0.5 mm that were white on the solid phase slope of the L-S StrepB, with no color change observed in the liquid phase (Fig. 2).

### Optimum time interpretations of L-S StrepB for the detection of GBS

A total of 87.3% (55/63) of GBS on L-S StrepB were detected within 15–24 h. Additionally, eight strains were identified after extending the incubation to 48 h. Among the results, 12.7% (8/63) of GBS were detected exclusively in the liquid phase, 22.2% (14/63) were detected solely in the solid phase, and 65.1% (41/63) were detected in both phases (Table 1).

**TABLE 1:** Detection of GBS at different time points by L-S StrepB

Different locations of L-S StrepB	Time of GBS positive detection		Total
	15–24 h	48 h (increased）	
Liquid phase	5	3	8
Solid phase	9	5	14
Liquid and solid phases	41	0	41
Total	55	8	63

### Detection performance analysis

Overall, 65 (11.2%) pregnant women were confirmed to have GBS colonization. Of these, 39 (6.7%) GBS strains were detected using blood agar plates, whereas an additional 26 cases were identified solely by L-S StrepB. In total, 63 (10.8%) GBS strains were detected by L-S StrepB, with two additional strains identified after the enriched liquid was subcultured onto blood agar plates. These two GBS strains grew on the L-S StrepB, forming atypical colonies that did not produce the characteristic orange to orange-red pigment and were classified as non-β-hemolytic GBS. Notably, among the specimens initially determined to be negative by L-S StrepB, only these two GBS strains were found to be positive on blood agar plates after subculturing, whereas all others remained negative. A total of 37 GBS strains were identified using both L-S StrepB and blood agar plates. The detection rate of GBS using L-S StrepB was significantly higher than that achieved using blood agar plates, with a statistically significant difference noted (*P* < 0.05).

The sensitivity of L-S StrepB and blood agar plate was 96.9% and 60%, respectively. Table 2 summarizes the relative advantages and disadvantages of L-S StrepB compared to the blood agar plate, including the following: sensitivity, specificity, PPV, NPV, causes of potential false negatives, approximate cost analysis per test, interpretation of results, and procedural time. The advantages of L-S StrepB include simultaneous liquid-phase enrichment and solid-phase culture, thereby eliminating the need for additional enrichment steps. Additionally, the distinct color change observed facilitates easy identification by laboratory personnel.

**TABLE 2:** Performance of various GBS detection methods^*a*^

	Samples positive/total (%/95% CI)	
	**L-S StrepB**	**Blood agar plate**
Sensitivity	63/65 (96.9%/89.3%–99.6%)	39/65 (60.0%/47.1%–72.0%)
Specificity	517/517 (100%/99.3%–100.0%)	517/517 (100.0%/99.3%–100.0%)
PPV	63/63 (100.0%/94.3%–100.0%)	39/39 (100%/91.0%–100.0%)
NPV	517/519 (99.6%/98.5%–99.9%)	517/543 (95.2%/93.4%–96.8%)
Cases of potential false negatives	Non-β-hemolytic GBS strains that do not produce the characteristic red-orange pigment cannot be detected by the L-S StrepB.	The results are significantly influenced by the skill level of the personnel. Inexperienced staff may easily overlook atypical forms of GBS. Additionally, the presence of mixed flora in the samples complicates the isolation of GBS.
Time	15–24 h, negative extended to 48 h	18–24 h, negative extended to 48 h
Approximate cost per media	$2.0	$1.0
Interpretation of results	The color change is distinct and easy to differentiate, facilitating quick identification. The integration of solid and liquid phases eliminates the need for separate enrichment and isolation processes.	Suspected GBS isolated on blood agar plate needs to be identified through the CAMP test, latex agglutination serological identification, or with the assistance of MALDI-TOF MS, among other methods.

^*a*^ Number of samples positive/total (%/95% CI).

## DISCUSSION

Our study found that the GBS colonization rate in the southern Zhejiang region could reach 11.2%, which is higher than that reported by Yang et al. (19). This difference also highlights the significant implications of this study. In China, GBS screening commonly involves direct detection using a blood agar plate, which may contribute to lower detection rates. Although extensive GBS screening has been practiced in the USA and Europe, direct blood agar plate is widely performed in China to reduce time and costs (5, 14, 20). However, when samples are heavily contaminated with other microorganisms, isolating the GBS strain becomes challenging, resulting in a high rate of false negatives, which places significant demands on technical staff. Research conducted in Korea has demonstrated that GBS colonization rates determined by culture assays vary across institutions, even within the same region (21). In this study, the sensitivity of the blood agar plate for detecting GBS was only 60.0%, rendering it unsuitable for effective screening. In a related study conducted in Beijing, Chen et al. (22) reported the GBS colonization rate of 5.17% among perinatal pregnant women by using a direct blood agar plate, which was significantly lower than the 9.2% reported in a follow-up study by Li et al. (17). A study conducted in Shandong Province, China, found that the GBS colonization rate of GBS using differential agar plates without pre-enrichment was 6.7%. The authors of the study acknowledged that the colonization rate was likely underestimated owing to the routine use of blood agar plates for cultivation (16).

Commercially available NAATs for GBS detection enhance the sensitivity, reduce the time required for culture, and provide faster results. Numerous recent studies have introduced rapid and highly sensitive molecular diagnostic methods for the detection of GBS (23, 24). Nevertheless, because of the need to process specimens uniformly, they do not significantly decrease the turnaround time for specimens and may exhibit slightly lower specificity than traditional culture methods. Therefore, these methods may not be feasible for small, grassroots healthcare facilities. An external quality assessment revealed that among 44 participating institutions, 8 required improvements, resulting in an error rate of 14.1% (25). Further enhancements in detection performance are necessary for certain laboratories. A recent CDC survey demonstrated that only 18.7% of laboratories reported using NAAT for GBS screening, which may be attributed to the increased costs associated with this technology and its suitability for large hospitals (26). Certainly, the detection rate of GBS is also influenced by sampling methods and other factors. For example, the use of Amies transport medium and flocked swabs can enhance detection rates, although we have not discussed these factors here. Our study represents one of the few investigations assessing the efficacy of liquid-solid biphasic differential medium for the detection of GBS colonization in antepartum women. While biphasic culture systems, like the Castañeda medium, have been established for decades, this study evaluates L-S StrepB that combines this biphasic principle with Granada-type color-based visualization, specifically designed for GBS detection. The L-S StrepB combines both liquid enrichment and solid agar, incorporating selective agents that inhibit the growth of interfering microorganisms, in accordance with CDC guidelines. In our study, to adhere to the gold standard of initial broth enrichment followed by culturing, we also subcultured the enriched broth from the liquid phase of the L-S StrepB negative results onto blood agar plates. Suspected colonies were subsequently verified by MALDI-TOF MS, further demonstrating the high sensitivity of the L-S StrepB. Although two more strains of non-hemolytic GBS were detected, we acknowledge that this approach cannot completely exclude false negatives. Potential limitations remain, including (i) undetected non-hemolytic strains due to technical factors, and (ii) the inherent detection threshold of culture-based methods. Future studies should incorporate NAAT or other phenotypic characterization to precisely determine sensitivity and specificity.

Similar to other Granada-type media, L-S StrepB enables specific detection of β-hemolytic GBS strains through granadaene pigment production, exhibiting distinctive orange-red colonies and enabling visual detection. This mechanism fundamentally differs from other chromogenic media, like ChromID StrepB, that rely on enzymatic cleavage of synthetic substrates. Granadaene is encoded by the cyl operon, which co-expresses with β-hemolysin, explaining why Granada-type media cannot detect non-hemolytic GBS strains. This is also a limitation shared with blood agar methods that require technical expertise for accurate interpretation. It is worth noting that, as granadaene is an important GBS virulence factor, non-β-hemolytic strains are less virulent and seldom cause neonatal disease (27, 28). However, the prevalence of non-β-hemolytic GBS is extremely low, accounting for only 3.1% in Zhejiang, which is negligible compared with the false-negative rate associated with the blood agar plate. Nevertheless, one study found that utilizing blood agar plates to identify non-pigmented colonies did not lead to a significant increase in the detection rate of GBS (14). Additional identification of non-pigmented colonies could be beneficial in regions and laboratories equipped with appropriate resources. Furthermore, this identification process is straightforward and user-friendly, allowing for inoculation and culture to be performed at any time and in any location. While the material cost of L-S StrepB is higher than conventional blood agar plates, its integrated color-based visual detection eliminates the need for subsequent identification steps, significantly reducing overall labor costs.

GBS was detected in 87.3% of the cases within 15–24 h using L-S StrepB, facilitating rapid reporting for clinical applications. Only 12.7% of GBS cases were detected solely in the liquid phase, which we hypothesize is due to the low concentration of GBS in the samples, preventing visible colony growth on the solid agar after selective enrichment. Conversely, 22.2% of GBS were detected exclusively on the solid phase, indicating that the colorimetric signal in the liquid phase was insufficient for visual recognition. The subculture of the broth into a blood agar plate confirmed GBS growth. If reliance is placed solely on the liquid medium, there is a risk that some positive samples may be overlooked. Therefore, the integration of both phases significantly enhances the overall sensitivity of the detection method.

In summary, L-S StrepB showed high sensitivity and specificity in our study cohort, though molecular experiments or other phenotypic experiments would further validate these findings. Additional evaluation of inter-laboratory variability remains essential to fully characterize its performance. Crucially, L-S StrepB achieved rapid mean detection times while maintaining user-friendly operation and cost-effectiveness. As a biphasic differential medium requiring no specialized equipment, it represents a practical solution for GBS screening in primary healthcare settings.

## References

[1] Brokaw A, Furuta A, Dacanay M, Rajagopal L, Adams Waldorf KM. 2021. Bacterial and host determinants of group b streptococcal vaginal colonization and ascending infection in pregnancy. Front Cell Infect Microbiol 11:720789. doi:10.3389/fcimb.2021.720789 34540718 PMC8446444

[2] Liu Y, Liu J. 2022. Group B Streptococcus: virulence factors and pathogenic mechanism. Microorganisms 10:2483. doi:10.3390/microorganisms10122483 36557736 PMC9784991

[3] Manuel G, Twentyman J, Noble K, Eastman AJ, Aronoff DM, Seepersaud R, Rajagopal L, Adams Waldorf KM. 2024. Group B streptococcal infections in pregnancy and early life. Clin Microbiol Rev. doi:10.1128/cmr.00154-22:e0015422 PMC11905376 39584819

[4] Stocker M, Rosa-Mangeret F, Agyeman PKA, McDougall J, Berger C, Giannoni E. 2024. Management of neonates at risk of early onset sepsis: a probability-based approach and recent literature appraisal : update of the Swiss national guideline of the Swiss Society of Neonatology and the Pediatric Infectious Disease Group Switzerland. Eur J Pediatr 183:5517–5529. doi:10.1007/s00431-024-05811-0 39417838 PMC11527939

[5] Anonymous. 2020. Prevention of group B streptococcal early-onset disease in newborns. Obstetrics & Gynecology 135:489–492. doi:10.1097/AOG.0000000000003669 31977793

[6] Society of Perinatal Medicine, Chinese Medical Association; Obstetrics Subgroup, Society of Obstetrics and Gynecology, Chinese Medical Association. 2021. Chinese experts consensus on prevention of perinatal group B streptococcal disease. Chin J Perinat Med 24(8):561–566. doi:10.3760/cma.j.cn113903-20210716-00638

[7] Verani JR, McGee L, Schrag SJ, Division of Bacterial Diseases, National Center for Immunization and Respiratory Diseases, Centers for Disease Control and Prevention (CDC). 2010. Prevention of perinatal group B streptococcal disease--revised guidelines from CDC, 2010. Vol. 59. 21088663

[8] Filkins L, Hauser JR. 2020. American Society for Microbiology provides 2020 guidelines for detection and identification of group B Streptococcus. J Clin Microbiol 59. doi:10.1128/JCM.01230-20 PMC7771461 33115849

[9] Church DL, Baxter H, Lloyd T, Larios O, Gregson DB. 2017. Evaluation of StrepBSelect chromogenic medium and the fast-track diagnostics group B Streptococcus (GBS) real-time PCR assay compared to routine culture for detection of GBS during antepartum screening. J Clin Microbiol 55:2137–2142. doi:10.1128/JCM.00043-17 28446575 PMC5483915

[10] Kfouri R de Á, Pignatari ACC, Kusano EJU, Rocchetti TT, Fonseca CL, Weckx LY. 2021. Capsular genotype distribution of group B Streptococcus colonization among at-risk pregnant women in Sao Paulo, Brazil. Braz J Infect Dis 25:101586. doi:10.1016/j.bjid.2021.101586 34081894 PMC9392176

[11] Huang J, Li S, Li L, Wang X, Yao Z, Ye X. 2016. Alarming regional differences in prevalence and antimicrobial susceptibility of group B streptococci in pregnant women: a systematic review and meta-analysis. J Glob Antimicrob Resist 7:169–177. doi:10.1016/j.jgar.2016.08.010 27837713

[12] Wang J, Zhang Y, Lin M, Bao J, Wang G, Dong R, Zou P, Chen Y, Li N, Zhang T, Su Z, Pan X. 2023. Maternal colonization with group B Streptococcus and antibiotic resistance in China: systematic review and meta-analyses. Ann Clin Microbiol Antimicrob 22:5. doi:10.1186/s12941-023-00553-7 36639677 PMC9837753

[13] Ge Y, Pan F, Bai R, Mao Y, Ji W, Wang F, Tong H. 2021. Prevalence of group B Streptococcus colonization in pregnant women in Jiangsu, East China. BMC Infect Dis 21:492. doi:10.1186/s12879-021-06186-5 34044786 PMC8161607

[14] Gao K, Deng Q, Huang L, Chang CY, Zhong H, Xie Y, Guan X, Liu H. 2021. Diagnostic performance of various methodologies for group B Streptococcus screening in pregnant woman in China. Front Cell Infect Microbiol 11:651968. doi:10.3389/fcimb.2021.651968 34109134 PMC8183470

[15] Wu LJ, Wang FL, Zou JH, Yang J, Huang YE, Ming F, Chen XR, Chen RR, Zhu YF. 2019. Analysis of screening strategy of group B Streptococcus in the third trimester and its influence on pregnancy outcome. Zhonghua Fu Chan Ke Za Zhi 54:154–159. doi:10.3760/cma.j.issn.0529-567x.2019.03.003 30893715

[16] Jiao J, Wu W, Shen F, Liu Z, Zhou H, Fan G, Zhou Y. 2022. Clinical profile and risk factors of group B Streptococcal colonization in mothers from the Eastern District of China. J Trop Med 2022:5236430. doi:10.1155/2022/5236430 36211624 PMC9534697

[17] Li Y, Yang W, Li Y, Hua K, Zhao Y, Wang T, Liu L, Liu Y, Wang Y, Liu W, Zhang L, Zhu R, Yu S, Sun H, Dou H, Yang Q, Xu Y, Guo L. 2024. The increasing burden of group B Streptococcus from 2013 to 20233: a retrospective cohort study in Beijing, China. Microbiol Spectr 13:e0226624. doi:10.1128/spectrum.02266-24 39656016 PMC11705810

[18] Ding Y, Wang Y, Hsia Y, Russell N, Heath PT. 2020. Systematic review and meta-analyses of incidence for group B Streptococcus disease in infants and antimicrobial resistance, China. Emerg Infect Dis 26:2651–2659. doi:10.3201/eid2611.181414 33079042 PMC7588546

[19] Yang L, Bao F, Wu Y, Sun L. 2020. Relationship of group B streptococcus colonization in late pregnancy with perinatal outcomes. Zhejiang Da Xue Xue Bao Yi Xue Ban 49:389–396. doi:10.3785/j.issn.1008-9292.2020.04.12 32762169 PMC8800799

[20] Di Renzo GC, Melin P, Berardi A, Blennow M, Carbonell-Estrany X, Donzelli GP, Hakansson S, Hod M, Hughes R, Kurtzer M, Poyart C, Shinwell E, Stray-Pedersen B, Wielgos M, El Helali N. 2015. Intrapartum GBS screening and antibiotic prophylaxis: a European consensus conference. J Matern Fetal Neonatal Med 28:766–782. doi:10.3109/14767058.2014.934804 25162923

[21] Sung JH, Cha HH, Lee NY, Lee WK, Choi Y, Han HS, Lee YY, Chong GO, Seong WJ. 2022. Diagnostic accuracy of loop-mediated isothermal amplification assay for group B Streptococcus detection in recto-vaginal swab: comparison with polymerase chain reaction test and conventional culture. Diagnostics (Basel) 12:1569. doi:10.3390/diagnostics12071569 35885476 PMC9322697

[22] Chen Y, Liu L, Liu J, Ji T, Gao Y, Yang D, Zhao M, Zhai Y, Cao Z. 2024. Serotype distribution, antimicrobial resistance, and molecular characterization of group B Streptococcus isolates from Chinese pregnant woman. J Matern Fetal Neonatal Med 37:2295805. doi:10.1080/14767058.2023.2295805 38124302

[23] Caballero Méndez A, Reynoso de la Rosa RA, Abreu Bencosme ME, Sosa Ortiz MN, Pichardo Beltré E, de la Cruz García DM, Piñero Santana NJ, Bacalhau de León JC. 2024. Development and performance evaluation of a qPCR-based assay for the fully automated detection of group B Streptococcus (GBS) on the panther fusion open access system. Microbiol Spectr 12:e0005724. doi:10.1128/spectrum.00057-24 38682931 PMC11237499

[24] Liu M, Wang H, Chu C, Min F, Sun L, Zhang T, Meng Q. 2024. Establishment and application of a rapid molecular diagnostic platform for the isothermal visual amplification of group B Streptococcus based on recombinase polymerase. Front Cell Infect Microbiol 14:1281827. doi:10.3389/fcimb.2024.1281827 38465235 PMC10920233

[25] Chen Y, Zhao R, Huang Z, Chu C, Xiao Y, Hu X, Wang X. 2024. A small-scale external quality assessment for PCR detection of group B Streptococcus in China. Clinica Chimica Acta 553:117733. doi:10.1016/j.cca.2023.117733 38128816

[26] Fay K, Almendares O, Robinson-Dunn B, Schrag S. 2019. Antenatal and intrapartum nucleic acid amplification test use for group B Streptococcus screening-United States, 2016. Diagn Microbiol Infect Dis 94:157–159. doi:10.1016/j.diagmicrobio.2018.11.026 30642719

[27] Rosa-Fraile M, Dramsi S, Spellerberg B. 2014. Group B streptococcal haemolysin and pigment, a tale of twins. FEMS Microbiol Rev 38:932–946. doi:10.1111/1574-6976.12071 24617549 PMC4315905

[28] Rosa-Fraile M, Spellerberg B. 2017. Reliable detection of group B Streptococcus in the clinical laboratory. J Clin Microbiol 55:2590–2598. doi:10.1128/JCM.00582-17 28659318 PMC5648696

